# Early hyperlactatemia predicts pancreatic fistula after surgery

**DOI:** 10.1186/s12871-015-0093-x

**Published:** 2015-07-28

**Authors:** Nicolas De Schryver, Xavier Wittebole, Catherine Hubert, Jean-François Gigot, Pierre-François Laterre, Diego Castanares-Zapatero

**Affiliations:** 1 Department of Critical Care Medicine, Cliniques Universitaires Saint Luc, Université Catholique de Louvain (UCL), Avenue Hippocrate 10, B-1200 Brussels, Belgium; 2 Department of Hepato-biliary Surgery, Cliniques Universitaires Saint Luc, Université Catholique de Louvain (UCL), Avenue Hippocrate 10, B-1200 Brussels, Belgium

**Keywords:** Pancreatic fistula, Hyperlactatemia, Pancreatic surgery

## Abstract

**Background:**

Postoperative pancreatic fistula (POPF) is a major complication after pancreatic surgery and results from an impaired healing of the pancreatic enteric anastomosis. Whether perioperative hemodynamic fluid management aiming to provide an adequate tissue perfusion could influence the occurrence of POPF is unknown. Serum lactate level is a well-recognized marker of decreased tissue perfusion and is known to be associated with higher morbidity and mortality in various postoperative settings. We aimed to determine in a retrospective high-volume center’s cohort whether postoperative hyperlactatemia could predict POPF occurrence.

**Method:**

We conducted a retrospective analysis of 96 consecutive patients admitted in the intensive care unit (ICU) after pancreaticoduodenectomy or distal pancreatectomy. Univariate analysis was conducted to compare lactate levels at 6 h between patients evolving with *versus* without POPF. A logistic regression model was developed and included potential confounding factors.

**Results:**

POPF occurred in 28 patients (29 %). Serum lactate level 6 h after admission was significantly higher in the POPF group (2.8 mmol/L [95 % confidence interval (CI): 2.1–3.5] *versus* 1.8 mmol/L [95 % CI: 1.8–2.4], *p*-value = 0.04) whereas it did not differ at ICU admission or at 12 h. Despite similar cumulative fluid balance, fluid intake and vasopressor use, hyperlactatemia > 2.5 mmol/L (Odds ratio (OR): 3.58; 95 % CI: 1.22–10.48; *p*-value = 0.020) and red blood cells transfusion (OR: 1.24; 95 % CI: 1.03–1.49; *p*-value = 0.022) were found to be independent predictive factors of POPF occurrence.

**Conclusion:**

In patients undergoing partial pancreatectomy, hyperlactatemia measured 6 h after ICU admission is a predictive factor for the occurrence of POPF. Inflammatory changes after surgery may account for this observation and should be further evaluated.

## Background

Pancreatic surgery is a complex procedure primarily performed for malignant diseases. Surgery remains the standard of care for pancreatic cancer and constitutes the sole treatment able to lengthen survival [1, 2]. Additionally, pancreatic surgery is also indicated for selected cases of chronic pancreatitis or less commonly for benign periampullary tumors [3–5]. While pancreaticoduodenectomy is chiefly used for pancreatic head resection, distal pancreactectomy is the preferred technique for lesions located in the pancreatic body or tail.

Though mortality has declined over the last decades, morbidity rates remain substantial, reaching 30 to 50 % even in high-volume centers [6–9]. Morbidity most commonly results from postoperative pancreatic fistula (POPF). POPF is caused by a deficient healing of the pancreatic-enteric anastomosis, leading to partial or complete anastomotic dehiscence. Described as the major complication following pancreatic surgery, its incidence has been reported to range from 5 to 35 % [10]. While a low-grade fistula is a transient leak that does not require specific treatment, externalized pancreatic juice may lead to life-threatening complications, such as intra-abdominal sepsis or hemorrhage. POPF substantially contributes to increased hospitalization length of stay, as percutaneous drainage of abdominal collection or surgical re-exploration is often required in these cases.

To date there has been no effective method for predicting the occurrence of fistula or for determining which will become clinically relevant. The diagnostic of fistula is still based on the amount of amylases in drains even if this method remains controversial [6, 10]. Numerous risk factors have been identified, which are mainl related to preoperative patient status and surgical procedures [7, 11–13]. Among the most relevant, pancreas texture, pancreatic duct diameter, blood loss, or comorbidities such as heart disease were shown to correlate with POPF. During the perioperative period, hypotension can reduce splanchnic perfusion, thereby compromising the healing of surgical anastomosis.

As a well-recognized hallmark of inadequate tissue perfusion, hyperlactatemia is common in critically ill patients and has been associated with mortality and morbidity in various clinical settings [14, 15]. Perioperative lactate levels, which frequently result from circulatory compromise, have been linked to clinical outcome in numerous surgical procedures [16–18]. Moreover, a relationship between hyperlactatemia and microcirculatory abnormalities has been demonstrated [19, 20]. Indeed, improvement in microcirculatory flow has been shown to be associated with decreased lactate levels [21]. Whether early postoperative hemodynamic management could play a role in POPF occurrence has not yet been studied. For this study, we hypothesized that early postoperative hyperlactatemia was related to POPF formation. For this purpose, we sought to determine whether hyperlactatemia predicts POPF occurrence after pancreatic surgery, along with its potential association with postoperative care.

## Methods

Following approval by the Cliniques universitaires Saint Luc’s Ethics committee, the medical records of 107 consecutive patients admitted, within three consecutive years, to the intensive care unit (ICU) after elective pancreatic surgery were reviewed. Due to the retrospective design of the study, informed consent was not indicated. To ensure confidentiality, patient identifiers were excluded from our final database. Of the 107 patients, 96 underwent partial pancreatectomy. Patients were excluded from the study if there was documentation of perioperative hemorrhagic shock. It was defined as the occurrence of massive intraoperative hemorrhage with estimated blood loss above 1000 mL, associated with the development of acute hemodynamic instability and anemia requiring transfusions of more than 5 red blood cells (RBC) units. Study measures included lactate levels, perioperative morbidity, hemodynamic and laboratory data within the first 24 h, cumulative 24-h fluid intake/balance, and length of ICU stay. Survival was recorded at 28 days, and morbidity was defined as a complication occurring within 30 days after surgical procedure or during hospital stay [22]. POPF was defined according to International Study Group of Pancreatic Fistula (ISGPF) definition as an amylase-positive fluid from the drain placed intraoperatively or from an abdominal collection (amylase levels >3 times the normal serum value) after the third day post-surgery [10].

Arterial plasma lactate levels were measured at ICU admission and at 6 and 12 h thereafter through a point-of-care blood gas analyzer.

Patients were grouped into those displaying hyperlactatemia >2.5 mmol/L and those with lactate levels <2.5 mmol/L 6 h after ICU admission. The cut-off of 2.5 mmol/L was chosen like in septic patients for whom microcirculatory abnormalities are reported to be associated with either intermediate (2–3.9 mmol/L) or high lactate (≥4 mmol/L) levels [23].

Administration of somatostatin was initiated at the beginning of surgery for all patients. Postoperative hemodynamic optimization was achieved following a goal-directed therapy protocol aiming at normalizing hemodynamic and perfusion parameters within the first 6 h. Circulatory and perfusion assessment included arterial lactate, mixed venous saturation, central venous-to-arterial O_2_ content_,_ and urinary output. Preset goals were lactate levels < 1.8 mmol/l, central venous oxygen saturation (ScVO2) >65 %, central venous-to-arterial O_2_ content <6 mlO_2_/dL, and central venous pressure >10 mmHg. Red blood cell (RBC) transfusions were used to maintain hemoglobin concentration above 7 g/dL.

### Statistical analysis

For continuous variables, mean (standard deviation) or median (interquartile range) were reported. The number of patients and the corresponding percentages in each category were given for categorical variables. Student’s t-test was used to compare continuous variables between subgroups. Mann–Whitney U test was employed when appropriate. Categorical variables were compared using Pearson’s Chi-squared test or Fisher’s exact test.

Univariate logistic regression analysis was conducted to compare several postoperative factors between patients evolving with *versus* without POPF. Logistic regression analysis stratified on the presence or absence of hyperlactemia at 6 h post-surgery was used to identify independent risk factors related to POPF occurrence. Significant variables in the univariate analysis were entered into a multivariate logistic regression to identify the independent predictors of fistula occurence. The variables were previously tested for interaction. Results were expressed as odds ratio and 95 % confidence intervals. Two-tailed p values of 0.05 were considered statistically significant. Analyses were performed using SPSS 20 software (IBM, Chicago, IL, USA).

## Results

Of the 96 patients with partial pancreatic surgery, 82 % underwent pancreaticoduodenectomy and 18 % distal pancreatectomy. Two patients were excluded due to perioperative hemorrhagic shock. The patients ranged in age from 27 to 82 years (median age: 65), and 59 % were men. Malignant disease was present in 78 patients (83 %). Overall 28-day mortality was 3.2 %. Body mass index (BMI) was significantly higher in patients evolving with POPF. Preoperative data is shown in Table 1.

**Table 1:** Patients characteristics and preoperative data

Patient characteristics	Overall (*n* = 94)	No fistula (*n* = 66)	Fistula (*n* =28)	*p*-value
Demographics				
age^a^	65 (27–82)	64 (27–82)	65.5 (40–80)	0.77
male^b^	56 (59)	38 (40)	18 (19)	0.55
Body mass index (kg/m^2^)^c^	25.4 (4.7)	24.7 (3.9)	27.5 (4.1)	0.04
Comorbidities				
hypertension^b^	19 (20.5 %)	11 (12 %)	8 (8.5 %)	0.18
diabetes mellitus^b^	17 (18 %)	12 (13 %)	5 (5 %)	0.97
ischemic cardiopathy^b^	15 (16 %)	11 (12 %)	4 (4 %)	0.99
Surgical procedures				
pancreaticoduodenectomy^b^	77 (82 %)	56 (60 %)	21 (22 %)	0.26
distal pancreatectomy^b^	17 (18 %)	10 (11 %)	7 (7 %)	0.26
Pathological diagnosis				
benign disease^b^	16 (17 %)	10 (11 %)	6 (6 %)	0.55
malignant disease^b^	78 (83 %)	56 (60 %)	22 (23 %)	0.55

^a^values expressed as median (range); *p*-value by Mann–Whitney U test

^b^values expressed as number and percentage; *p*-value by χ^2^ test or exact Fisher test

^c^values expressed as mean ± standard deviation; *p*-value by Student t test

In total, 35 % of surgical complications were encountered (*n* = 33 patients). Of these, POPF occurred in 28 patients (29 %) classified as Grade A (*n* = 11), Grade B (*n* = 9), or Grade C (*n* = 8). Patients with Grade A fistula required no specific treatment, whereas patients with Grade B fistula were treated with conservative treatment including maintenance of peripancreatic drains, antibiotherapy, parenteral nutrition, and somatostatine. Patients with Grade C fistula experienced complications such as intraabdominal collections requiring drainage (*n* = 8), anastomotic failure (*n* = 3), and/or hemorrhage (*n* = 7). Six patients with POPF developed sepsis. Surgical revision was necessary for 14 patients, six of whom had POPF. The incidence of each complication is shown in Table 2.

**Table 2:** Surgical complications

Complication^a^	*n* (%)
pancreatic fistula	28 (29 %)
intraabdominal collections	11 (12 %)
hemorrhage	3 (3.2 %)
anastomotic failure	2 (2.1 %)
wound infection	3 (3.2 %)
bile leakage	3 (3.2 %)
portal vein thrombosis	4 (4.2 %)
other^b^	2 (2.1 %)

^a^Some patients developed multiple complications

^b^Other complications included small bowel perforation (*n* =1) and mesenteric ischemia (*n* =1)

As detailed in Table 3, postoperative lactate serum concentrations at 6 h were significantly higher in the POPF group, whereas no difference was observed at baseline (on ICU admission) and at 12 h post-ICU admission. Further, the proportion of patients presenting lactate levels above 2.5 mmol/L at 6 h was significantly higher in the POPF group. Both groups were comparable regarding use of norepinephrine and its cumulative dose, along with the amount of infused liquid intraoperatively and postoperatively. Additionally, fluid balances during the first 24 h, APACHE II scores and intervention durations were similar (Table 3). However, the amount of RBC transfusions during the first 24 postoperative hours was significantly higher in the POPF group. Nevertheless, hemoglobin levels measured at 24 h were comparable. Finally, mean ScVO_2_ values were similar in both subgroups at admission (67 % [65–73] *versus* 65 % [56–79]) and over the first 6 h (67 % [57–70] *versus* 66 % [61–71]) and 12 h (65 % [58–68] *versus* 65 % [55–68]) following ICU admission.

**Table 3:** Postoperative data

	No Fistula (*n* = 66)	Fistula (*n* =28)	*p* value
Postoperative baseline lactate (mmol/L)^a^	1.9 (1.8)	2.1 (1.6)	0.64
6 h postoperative lactate (mmol/L)^a^	1.8 (1.3)	2.8 (2.5)	0.04
12 h postoperative lactate (mmol/L)^a^	1.7 (1.1)	1.8 (1.4)	0.81
Hyperlactatemia (blood lactate ≥ 2.5 mmol/L)^b^	18 (27 %)	17 (60 %)	0.002
Vasopressor use^b^	8 (13 %)	7 (25 %)	0.13
Cumulative norepinephrine dose (μg/kg)^a^	57 (43)	48 (12)	0.18
Fluid intake during first 24 h (mL/kg)^a^	46.2 (34)	48.1 (43)	0.15
Fluid balance during first 24 h (mL)^a^	2160 (2000)	2631 (2339)	0.34
Intraoperative fluid requirements (mL/kg)^a^	52.6 (25)	49 (22)	0.39
Red blood cells transfusions during first 24 h (mL)^a^	164 (250)	359 (530)	0.027
Hemoglobin after first 24 h (g/dL)^c^	10.4 ± 1.8	10.8 **±** 1.5	0.35
APACHE II score^a^	12 (6)	12.5 (8)	0.98
Duration of operation (minutes)^c^	356 ± 96	316 ± 94	0.91

^a^data are shown as median (interquartile range); *p*-value by Mann–Whitney U test

^b^data are shown as number (percentage); *p*-value by χ^2^ test or exact Fisher test

^c^data are shown as mean ± standard deviation; *p*-value by Student t test

In univariate analysis, hyperlactatemia, use of norepinephrine, RBC transfusions, and BMI were identified as significant risk factors associated with POPF occurrence (Table 4). Fluid intake or fluid balance did not impact POPF development.

**Table 4:** Risk factors associated with postoperative pancreatic fistula following pancreaticoduodenectomy

	Univariate		Multivariate	
	Odds ratio (95 % CI)	*p*-value	Odds ratio (95 % CI)	*p*-value
Demographics				
Body mass index	1.15 (1.10–1.27)	0.015^*^	1.11 (0.99–1.25)	0.085
Ischemic cardiopathy	1.18 (0.32–4.32)	0.79		
Post-operative variables				
Hyperlactatemia	4.36 (1.70–11.15)	0.002^*^	3.58 (1.22–10.48)	0.020^*^
RBC transfusions	1.23 (1.05–1.45)	0.008^*^	1.24 (1.03–1.49)	0.022^*^
Hemoglobin (24 h)	1.13 (0.87–1.47)	0.35		
Vasopressor use	4.10 (1.14–13.97)	0.003^*^	2.10 (0.47–9.28)	0.33
Fluid intake (24 h)	1.01 (0.99–1.03)	0.12		
Fluid balance (24 h)	1.19 (0.61–2.33)	0.59		

^*^denotes *p* < 0.05

Multiple regression analysis showed that hyperlactatemia at 6 h and RBC transfusions were still significant independent predictors of POPF occurrence, whereas noradrenaline use or BMI were no longer significant predictors in the final model (Table 4). As hyperlactatemia occurred independently of RBC transfusions and hemodynamic achievements, we tried to determine whether hyperlactatemia was inflammation-mediated. To assess whether the systemic inflammatory response following surgery differed in both group, C-reactive protein (CRP) was evaluated. Mean CRP values were significantly higher in the POPF group (14.2 ± 8 *versus* 10 ± 4.7; *p* = 0.02).

## Discussion

In our study, we found that hyperlactatemia after pancreatic surgery is associated with a risk of POPF development, but is independent of early-ICU postoperative hemodynamic management. Indeed, intravenous fluid or vasopressive therapies were not related to POPF occurrence in the adjusted regression model. Both patient groups received approximately similar amounts of intravenous fluid, and the need for vasopressors was similar. Although this study was only hypothesis-generating, our data supports the view that patients exhibiting hyperlactatemia 6 h after the initial ICU management show an association with a distinctive clinical profile despite similar postoperative care.

Hyperlactatemia can reflect global or regional hypoperfusion, which may be responsible for an impaired healing of the anastomosis, leading to POPF. Lactic acid is produced from the transformation of pyruvate by the lactate dehydrogenase enzyme when anaerobic metabolism is favored due to a lack of oxygen supply to the tissues [24]. Peripheral tissue hypoperfusion is frequently encountered after major abdominal surgery and is associated with postoperative complications [25] and more particularly pancreatic fistula [7]. Per- and post-operative management mainly aims at restoring intravascular volume and global hemodynamics in order to assure adequate tissue perfusion [26, 27]. Moreover, it has been shown to influence postoperative complications. In this settings, hyperlactatemia as a marker of global hypoperfusion is well known to correlate with surgical complications and mortality [28, 29].

In our study, postoperative management was achieved by aiming to normalize hemodynamic and perfusion parameters within 6 h after ICU admission. As serum lactate levels were higher in the group that developed POPF, it could be argued that these patients were more hypotensive and fluid resuscitation less adequate. However, we did not notice any differences in the global hemodynamic of these patients. Indeed, the amounts of norepinephrine required postoperatively were quite similar, as were total fluid amounts administrated. Moreover, the circulatory parameters (mixed venous saturation; central venous-to-arterial O_2_ content), urinary output, and fluid balance did not differ between the two groups. This suggests that hemodynamic management was similar in both groups and could thus not be the cause of POPF occurrence. In addition, infused intravenous fluids amounts during surgery did not differ between both groups. Even if patients experiencing POPF received more RBC transfusions, hemoglobin 24 h after surgery was ultimately similar in both groups. Therefore, multivariate analysis revealed RBC transfusion to be an independent risk factor for POPF. This is in line with previous work which demonstrated that postoperative bleeding was associated with POPF occurrence [30].

Although lower oxygen delivery can be responsible for anastomotic ischemia, it cannot solely explain hyperlactatemia, which also represented an independent risk factor in the multivariate model.

Lactate may be generated by inflammation in relation with the systemic inflammatory response following critical illness [31]. It is associated with outcome independently of shock in various diseases, such as sepsis or after surgery [17–20]. In addition, lactate levels have been demonstrated to be related to microcirculatory perfusion alterations and organ failure [21–23]. Hyperlactatemia could thus reflect the inflammation-induced microcirculatory alterations following surgery. Indeed, surgery induces a high inflammatory response reflected by increased cytokine production. Cytokines such as interleukin-6 have been shown to play a central role [32]. A recent study has demonstrated that intraperitoneal lactate levels after pancreatic surgery were higher in patients developing POPF than in those in whom no complication occurred [33]. This suggests that microcirculatory disturbances consequently leading to local ischemia may be a determinant in POPF occurrence. Nevertheless, the authors observed increased lactate/pyruvate ratio within the peritoneum without any difference in serum lactate levels. In their study, lactate was measured 12 h after surgery which is later than our study.

The higher CRP levels observed in our study in the POPF group tend to suggest inflammatory response following surgery to be associated with POPF. Postoperative inflammatory response has indeed been shown to correlate with poor outcome in patients undergoing pancreatic surgery [34]. Other data has also revealed that CRP levels can predict poorer prognosis [35]. It can therefore be suggested that, independently of basal inflammation related to cancer, surgery could generate additional inflammation. In this setting, although 6 h-postoperative lactate values are significantly higher in the POPF group, the values decreased 12 h after surgery and were similar within both groups. It is worth noting that lactate values are elevated in both groups at admission (Table 3) but the increase is more important in patients developing fistula. This difference between kinetics could reflect a distinct evolution in the time course of postoperative systemic inflammatory response syndrome (SIRS) and particularly in the early phase. The mechanism by which hyperlactatemia occurs remains to be determined but a distinct profile of early SIRS should be considered and further investigated in patients experiencing fistula [36, 37].

Some limitations of our study should be considered. First, since we used a retrospective design, our results must be confirmed in a larger prospective cohort. Yet the use of a goal-oriented protocol in our unit allows us to trust our conclusion. Moreover, our observations are consistent with previously reported prospective data showing that pre- and post-operative serum lactate levels after pancreatic resection were correlated with ICU length of stay and mortality [29]. Second, we did not take into account the texture of pancreas tissue. Indeed, soft texture of pancreatic tissue encountered in malignant tumor has been shown to be associated with a higher POPF incidence after surgery. For this purpose, multivariate analysis was carried out, introducing adenocarcinoma as a covariable. When controlling for the presence of adenocarcinoma, the hyperlactatemia remained a risk factor for POPF. However, retrospectively collecting data regarding the pancreas texture was difficult. Lastly, we did not measure intraabdominal pressure. It is conceivable that high abdominal pressure encountered in obese patients could impair the anastomotic vascularisation. In addition, obesity has been recognized to be associated with a fatty texture of the pancreatic tissue and represents a risk factor for POPF [38, 39]. We found a correlation between BMI and POPF but it did not reach statistical significance in multivariate analysis.

## Conclusions

In summary, postoperative hyperlactatemia above 2.5 mmol/L was associated with POPF occurrence after pancreatic surgery independently of early hemodynamic management and RBC needs. This finding especially suggests a role for inflammatory changes that may account for the onset of anastomotic leakage after pancreatic surgery. According to our data, hyperlactatemia could be viewed as a surrogate marker for microcirculatory alterations leading to anastomotic ischemia. Confirmation of such a hypothesis would require further prospective investigation.

## References

[1] Shaib Y, Davila J, Naumann C, El-Serag H (2007). The impact of curative intent surgery on the survival of pancreatic cancer patients: a U.S. Population-based study. Am J Gastroenterol.

[2] Balcom JH, Rattner DW, Warshaw AL, Chang Y, Fernandez-del CC (2001). Ten-year experience with 733 pancreatic resections: changing indications, older patients, and decreasing length of hospitalization. Arch Surg.

[3] Fernández-del Castillo C, Morales-Oyarvide V, McGrath D, Wargo JA, Ferrone CR, Thayer SP (2012). Evolution of the Whipple procedure at the Massachusetts General Hospital. Surgery.

[4] Yeo CJ, Cameron JL, Sohn TA, Lillemoe KD, Pitt HA, Talamini MA (1997). Six hundred fifty consecutive pancreaticoduodenectomies in the 1990s: pathology, complications, and outcomes. Ann Surg.

[5] Sauvanet A (2002). Functional results of pancreatic surgery. Rev Prat.

[6] Lermite E, Sommacale D, Piardi T, Arnaud JP, Sauvanet A, Dejong CH (2013). Complications after pancreatic resection: diagnosis, prevention and management. Clin Res Hepatol Gastroenterol.

[7] Butturini G, Daskalaki D, Molinari E, Scopelliti F, Casarotto A, Bassi C (2008). Pancreatic fistula: definition and current problems. J Hepatobiliary Pancreat Surg.

[8] Ho CK, Kleeff J, Friess H, Büchler MW (2005). Complications of pancreatic surgery. HPB (Oxford).

[9] DeOliveira ML, Winter JM, Schafer M, Cunningham SC, Cameron JL, Yeo CJ (2006). Assessment of complications after pancreatic surgery: a novel grading system applied to 633 patients undergoing pancreaticoduodenectomy. Ann Surg.

[10] Bassi C, Dervenis C, Butturini G, Fingerhut A, Yeo C, Izbicki J (2005). Postoperative pancreatic fistula: an international study group (ISGPF) definition. Surgery.

[11] Seeliger H, Christians S, Angele MK, Kleespies A, Eichhorn ME, Ischenko I (2010). Risk factors for surgical complications in distal pancreatectomy. Am J Surg.

[12] Cheng Q, Zhang B, Zhang Y, Jiang X, Zhang B, Yi B (2007). Predictive factors for complications after pancreaticoduodenectomy. J Surg Res.

[13] Lermite E, Pessaux P, Brehant O, Teyssedou C, Pelletier I, Etienne S (2007). Risk factors of pancreatic fistula and delayed gastric emptying after pancreaticoduodenectomy with pancreaticogastrostomy. J Am Coll Surg.

[14] Husain FA, Martin MJ, Mullenix PS, Steele SR, Elliott DC (2003). Serum lactate level and base deficit as predictors of mortality and morbidity. Am J Surg.

[15] Trezeciak S, Dellinger RP, Chansky ME, Arnold RC, Schorr C, Milcarek B (2007). Serum lactate as predictor of mortality in patients with infection. Intensive Care Med.

[16] McNelis J, Marini CP, Jurkiewicz A, Szomstein S, Simms HH, Ritter G (2001). Prolonged lactate clearance is associated with increased mortality in the surgical intensive care unit. Am J Surg.

[17] Watanabe I, Mayumi T, Arishima T, Takahashi H, Shikano T, Nakao A (2007). Hyperlactemia can predict the prognosis of liver resection. Shock.

[18] Kogan A, Preisman S, Bar A, Sternik L, Lavee J, Malachy A (2012). The impact of hyperlactatemia on postoperative outcome after adult cardiac surgery. J Anesth.

[19] Yeh YC, Wang MJ, Chao A, Ko WJ, Chan WS, Fan SZ (2013). Correlation between early sublingual small vessel density and late blood lactate level in critically ill surgical patients. J Surg Res.

[20] Tripodaki ES, Tasoulis A, Koliopoulou A, Vasileiadis I, Vastardis L, Giannis G (2012). Microcirculation and macrocirculation in cardiac surgical patients. Crit Care Res Pract.

[21] Hernandez G, Boerma EC, Dubin A, Bruhn A, Koopmans M, Edul VK (2013). Severe abnormalities in microvascular perfused vessel density are associated to organ dysfunctions and mortality and can be predicted by hyperlactatemia and norepinephrine requirements in septic shock patients. J Crit Care.

[22] Dindo D, Demartines N, Clavien PA (2004). Classification of surgical complications: a new proposal with evaluation in a cohort of 6336 patients and results of a survey. Ann Surg.

[23] Mikkelsen ME, Miltiades AN, Gaieski DF, Goyal M, Fuchs BD, Shah CV (2009). Serum lactate is associated with mortality in severe sepsis independent of organ failure and shock. Crit Care Med.

[24] Fall PJ, Szerlip HM (2005). Lactic acidosis: from sour milk to septic shock. J Intensive Care Med.

[25] van Genderen ME, Paauwe J, de Jonge J, van der Valk RJ, Lima A, Bakker J (2014). Clinical assessment of peripheral perfusion to predict postoperative complications after major abdominal surgery early: a prospective observational study in adults. Crit Care.

[26] Scheeren TW, Wiesenack C, Gerlach H, Marx G (2013). Goal-directed intraoperative fluid therapy guided by stroke volume and its variation in high-risk surgical patients: a prospective randomized multicentre study. J Clin Monit Comput.

[27] Futier E, Constantin JM, Petit A, Chanques G, Kwiatkowski F, Flamein R (2010). Conservative vs restrictive individualized goal-directed fluid replacement strategy in major abdominal surgery: a prospective randomized trial. Arch Surg.

[28] Li S, Peng K, Liu F, Yu Y, Xu T, Zhang Y (2013). Changes in blood lactate levels after major elective abdominal surgery and the association with outcomes: a prospective observational study. J Surg Res.

[29] Gruttadauria S, Marino IR, Vitale CH, Mandala L, Scott VL, Doria C (2002). Correlation between peri-operative serum lactate levels and outcome in pancreatic resection for pancreatic cancer, preliminary report. J Exp Clin Cancer Res.

[30] Ricci C, Casadei R, Buscemi S, Minni F (2012). Late postpancreatectomy hemorrhage after pancreaticoduodenectomy: is it possible to recognize risk factors?. JOP.

[31] Gutierrez G, Williams JD (2009). The riddle of hyperlactatemia. Crit Care.

[32] Jawa RS, Anillo S, Huntoon K, Baumann H, Kulaylat M (2011). Analytic review: interleukin-6 in surgery, trauma, and critical care: part I: Basic Science. J Intensive Care Med.

[33] Ansorge C, Regner S, Segersvärd R, Strömmer L (2012). Early intraperitoneal metabolic changes and protease activation as indicators of pancreatic fistula after pancreaticoduodenectomy. Br J Surg.

[34] Jamieson NB, Glen P, McMillan DC, McKay CJ, Foulis AK, Carter R (2005). Systemic inflammatory response predicts outcome in patients undergoing resection for ductal adenocarcinoma head of pancreas. Br J Cancer.

[35] Szkandera J, Stotz M, Absenger G, Stojakovic T, Samonigg H, Kornprat P (2014). Validation of C-reactive protein levels as a prognostic indicator for survival in a large cohort of pancreatic cancer patients. Br J Cancer.

[36] Reisinger KW, Poeze M, Hulsewé KW, van Acker BA, van Bijnen AA, Hoofwijk AG (2014). Accurate prediction of anastomotic leakage after colorectal surgery using plasma markers for intestinal damage and inflammation. J Am Coll Surg.

[37] van der Voort PH, Westra B, Wester JP, Bosman RJ, van Stijn I, Haagen IA (2014). Can serum L-lactate, D-lactate, creatine kinase and I-FABP be used as diagnostic markers in critically ill patients suspected for bowel ischemia. BMC Anesthesiol.

[38] Ramsey AM, Martin RC (2011). Body mass index and outcomes from pancreatic resection: a review and meta-analysis. J Gastrointest Surg.

[39] Gaujoux S, Cortes A, Couvelard A, Noullet S, Clavel L, Rebours V (2010). Fatty pancreas and increased body mass index are risk factors of pancreatic fistula after pancreaticoduodenectomy. Surgery.

